# Near‐infrared photoimmunotherapy targeting EGFR—Shedding new light on glioblastoma treatment

**DOI:** 10.1002/ijc.31246

**Published:** 2018-01-19

**Authors:** Thomas A. Burley, Justyna Mączyńska, Anant Shah, Wojciech Szopa, Kevin J. Harrington, Jessica K.R. Boult, Anna Mrozek‐Wilczkiewicz, Maria Vinci, Jeffrey C. Bamber, Wojciech Kaspera, Gabriela Kramer‐Marek

**Affiliations:** ^1^ Division of Radiotherapy and Imaging The Institute of Cancer Research London United Kingdom; ^2^ Department of Neurosurgery Medical University of Silesia, Regional Hospital Sosnowiec Poland; ^3^ A. Chelkowski Institute of Physics, University of Silesia Katowice Poland; ^4^ Department of Onco‐Hematology Bambino Gesù Children's Hospital Rome Italy

**Keywords:** photoimmunotherapy, affibody molecules, glioblastoma, EGFR

## Abstract

Glioblastomas (GBMs) are high‐grade brain tumors, differentially driven by alterations (amplification, deletion or missense mutations) in the epidermal growth factor receptor (EGFR), that carry a poor prognosis of just 12–15 months following standard therapy. A combination of interventions targeting tumor‐specific cell surface regulators along with convergent downstream signaling pathways may enhance treatment efficacy. Against this background, we investigated a novel photoimmunotherapy approach combining the cytotoxicity of photodynamic therapy with the specificity of immunotherapy. An EGFR‐specific affibody (Z_EGFR:03115_) was conjugated to the phthalocyanine dye, IR700DX, which when excited with near‐infrared light produces a cytotoxic response. Z_EGFR:03115_–IR700DX EGFR‐specific binding was confirmed by flow cytometry and confocal microscopy. The conjugate showed effective targeting of EGFR positive GBM cells in the brain. The therapeutic potential of the conjugate was assessed both *in vitro*, in GBM cell lines and spheroids by the CellTiter‐Glo® assay, and *in vivo* using subcutaneous U87‐MGvIII xenografts. In addition, mice were imaged pre‐ and post‐PIT using the IVIS/Spectrum/CT to monitor treatment response. Binding of the conjugate correlated to the level of EGFR expression in GBM cell lines. The cell proliferation assay revealed a receptor‐dependent response between the tested cell lines. Inhibition of EGFRvIII+ve tumor growth was observed following administration of the immunoconjugate and irradiation. Importantly, this response was not seen in control tumors. In conclusion, the Z_EGFR:03115_–IR700DX showed specific uptake *in vitro* and enabled imaging of EGFR expression in the orthotopic brain tumor model. Moreover, the proof‐of‐concept *in vivo* PIT study demonstrated therapeutic efficacy of the conjugate in subcutaneous glioma xenografts.

## Abstract 2

**What's new?**

Photoimmunotherapy combines the destructive power of photodynamic therapy with the specificity of immunotherapy. But monoclonal antibodies bound to photosensitizers are large, and difficult to get deep into the tumor. Here, the authors attach a photosensitive dye to tiny “affibody” ligands designed to seek out EGFR, which is often overexpressed in glioblastoma. The affibody molecule brings the dye molecules into the cancer cells, and when it is exposed to near‐infrared wavelengths of light. It kills the cell. They showed that the EGFR‐affibody combined with the photoreactive dye selectively kills glioblastoma cells overexpressing EGFR in mice.

Glioblastoma (GBM) is a primary neuroepithelial tumor of the central nervous system, characterized by extremely aggressive clinical behavior driven by inter‐ and intrapatient genomic and histopathological diversity.^1^ Patients with GBM have a poor prognosis, with a median overall survival of only 12–15 months and a 5‐year survival rate of 3–5%.^2^ Currently, standard‐of‐care treatment includes maximal tumor resection followed by radiotherapy with concomitant and adjuvant systemic therapies, for example, temozolomide.^3^ The presence of residual tumor cells post‐surgery for high‐grade gliomas has been reported in 65% of cases and is one of the major factors contributing to GBM recurrence.^4^, ^5^ Several groups have demonstrated that patients with gross total resection in the setting of low‐ and high‐grade gliomas have improved survival rates compared to patients undergoing subtotal resection.^6^, ^7^ Therefore, real‐time intraoperative treatment strategies are urgently needed to improve surgical outcomes. Fluorescence‐guided surgery (FGS) combined with photodynamic therapy (PDT) is an interesting approach that may allow simultaneous visualization and treatment of infiltrating tumor cells. Recently, a number of fluorescent markers, including fluorescein sodium, indocyanine green (ICG) and photosensitizers (PSs), such as Photofrin, Talaporfirin sodium and 5‐aminolevulinic acid (5‐ALA), have been validated in clinical trials for intraoperative delineation of tumor boundaries to facilitate maximal safe resection and/or eradiation of infiltrative tumor cells.^8^, ^9^, ^10^ For instance, Stummer *et al*. have shown that patients who underwent FGS with 5‐ALA had significantly improved gross total resection (65% *vs*. 36%; *p* <0.0001) and 6‐month progression‐free survival (41% *vs*. 21%; *p* <0.0003) rates compared to patients that underwent conventional microsurgery under white light.^4^ In addition, Eljamel *et al*. have reported that FGS and repetitive Photofrin or 5‐ALA‐based PDT significantly improved median overall (12.2 *vs*. 5.6 months for the control group) and progression‐free survival (8.6 *vs*. 4.8 months) in a Phase III randomized controlled trial in patients with GBM.^11^ Even though these PSs have been used as diagnostic and therapeutic agents, they lack specific cancer cell‐targeting potential and, due to their low absorption coefficients, suffer from poor activation sensitivity.^12^ Therefore, developing novel, near‐infrared (NIR) molecularly targeted probes for intraoperative treatment of high‐grade gliomas is an attractive prospect.^13^, ^14^

In recent years, there has been considerable interest surrounding antibody‐based PDT, also called photoimmunotherapy (PIT), which utilizes the targeting ability of a highly specific monoclonal antibody (mAb) conjugated to a PS. Following excitation with NIR light, the PS generates reactive oxygen species (ROS), which cause cytotoxic effects exclusively in cancer cells aberrantly overexpressing the target receptors and sparing adjacent normal tissues.^15^ Conversely, conventional PDT, while effective in inducing cell death, employs non‐targeted PSs that affect non‐cancerous cells, relying entirely on physical targeting by localized optical irradiation, which limits their clinical application due to side effects (e.g., cutaneous photosensitivity).^16^ Accordingly, PIT applied during GBM surgery could be used as an adjuvant strategy, allowing the surgeon not only to visualize fluorescently positive margins or microscopic residual lesions, but also to eradicate residual or surgically inaccessible tumor cells.

Amplification of the epidermal growth factor receptor (EGFR) is the commonest genetic aberration, occurring in about 50% of *de novo* primary GBMs, half of which harbor the EGFRvIII mutation (in‐frame deletion of exons 2–7) leading to constitutive and ligand‐independent receptor activity.^17^ Thus, there is a strong rationale to develop an EGFR‐targeted PIT strategy, guided by functional molecular imaging, which could significantly improve GBM patient management.

Among all of the clinically useful PSs, the phthalocyanine IRDye700DX (hereafter called IR700DX) seems to have the most favorable chemical properties. The dye is considerably less sensitive to photobleaching than many other fluorochromes, has excellent water solubility and can be covalently conjugated to a targeted molecule via an *N*‐hydroxysuccinimide ester or maleimide.^15^ Recent *in vitro* studies have demonstrated that IR700DX‐based mAb conjugates are highly specific for cells that express the target antigen, and have no effect on adjacent non‐expressing cells.^15^, ^18^ It has been found that, when the conjugate selectively binds to a target on the cell membrane and is exposed to NIR light, it induces rapid alterations in the cell membrane that ultimately lead to cell death.^18^ These promising preclinical findings have resulted in clinical trial initiation for the IR700DX–cetuximab conjugate, currently in a Phase I study in inoperable squamous cell carcinomas of the head and neck [NCT02422979]. In line with these findings, Ogawa and coworkers have reported that this process promotes the relocation of immunogenic cell death markers (e.g., calreticulin, Hsp90) to the cell membrane and subsequent release of immunogenic signals including ATP and HMGB1.^19^

While mAb‐based immunoconjugates offer exquisite selectivity of binding to their designated targets, their poor extravasation into the tumor (due to their relatively large molecular size) hampers penetration into the tumor's parenchyma, markedly limiting the effectiveness of therapy. Therefore, to circumvent this concern, we have developed an IR700DX‐based conjugate using low molecular weight (∼7 kDa) EGFR‐specific affibody molecules as our targeting moiety (Z_EGFR:03115_–IR700DX). The lack of disulfide bonds and internal cysteines, rapid folding properties and high stability of affibody molecules facilitate their conjugation with different radionuclides or fluorophores.^20^ Moreover, the high binding affinity (pM to nM range) of these molecules to wild‐type EGFR, as well as EGFRvIII, their small size (resulting in rapid clearance from the circulation with predominantly renal excretion *in vivo*), and good tumor penetration make them ideal targeting agents for GBM therapy.^21^

Herein, we demonstrate that administration of an affibody‐infrared light‐activated conjugate targeting EGFR selectively induces cell death in EGFR+ve GBM cells, while limiting toxicity in normal tissues.

## Material and Methods

### Reagents

The EGFR‐targeted affibody (Z_EGFR:03115_‐Cys) and the non‐specific affibody (Z_Taq_) were provided through our ongoing collaboration with AffibodyAB (Stockholm, Sweden). The hydrophilic IR700DX–maleimide used for affibody conjugation, IR700DX–NHS ester, IR700DX–carboxylate and IR800CW–maleimide were purchased from LI‐COR Biosciences (Lincoln, NE).

### Cell lines and cell culture

The GBM cell lines U87‐MG and U87‐MGvIII were kindly provided by Dr Frank Furnari (Ludwig Cancer Research, San Diego, CA) and maintained as previously described.^22^ The U251 cell line was courtesy of Prof. Chris Jones (The Institute of Cancer Research, London, UK). The WSz4 primary‐patient‐derived cell line was recently established in our lab (detailed method is provided in the Supporting Information). The breast cancer cell line MCF7 was obtained from the American Type Tissue Culture Collection (ATCC, Manassas, VA) within the last 6 months and cultured in DMEM (Gibco, Life Technologies) supplemented with 10% heat‐inactivated fetal bovine serum (FBS; Gibco, Life Technologies, Carlsbad, CA). Spheroids were generated as previously described.^23^ All cell lines were grown at 37°C in a humidified atmosphere containing 5% CO_2_.

### Conjugation of the affibody molecules with IR700DX

The conjugation of Z_EGFR:03115_ and Z_Taq_ to IR700DX–maleimide are described in detail in the Supporting Information.

### Western blotting

Western blotting was performed as previously described.^24^ For antibody details, see the Supporting Information.

### Flow cytometry

The specificity of the Z_EGFR:03115_–IR700DX binding *in vitro* was investigated using flow cytometry. A detailed description of the protocol and data analysis is given in the Supporting Information.

### Confocal microscopy

U251, U87‐MGvIII and MCF7 cells were plated onto confocal glass‐bottomed dishes (MatTek, Ashland, MA) at 2 × 10^5^ cells/dish and incubated for 24 h. For the 3D U87‐MGvIII or WSz4 cultures, cells (4 × 10^3^) were first seeded in 96‐well ultra‐low attachment plates (Corning® Costar®, Corning, NY) for 72 or 120 h and then transferred to confocal glass‐bottomed dishes. To test the specificity of conjugate binding, Z_EGFR:03115_–IR700DX (1 µM) or IR700DX alone (1 µM) were added to the medium and cells were incubated for 1, 3 or 6 h at 37°C. To analyse the penetration of the conjugate in comparison to an antibody‐based conjugate, U87‐MGvIII spheroids were incubated with either Z_EGFR:03115_–IR700DX (500 nM), anti‐EGFR‐targeted antibody‐FITC (500 nM) or IR700DX–maleimide alone (500 nM). Detailed descriptions of the procedures and image acquisition are described in the Supporting Information.

### Immunohistochemistry and fluorescence *ex vivo* imaging

Formalin‐fixed patient‐derived tumor samples, spheroids and excised U87‐MGvIII tumors, either following irradiation or affibody‐based PIT, were embedded in paraffin, sectioned (5 μm‐thick slices) and mounted on microscope slides. Multiple sections were taken at regular intervals across each tumor, with sequential sections being stained with H&E (Leica biosystems, Buffalo Grove, IL), anti‐Ki67 mAb (1:400, Cell Signaling Technology, Danvers, Massachusetts) and anti‐EGFR mAb (1:400, Dako, Santa Clara, CA). Orthotopic tumors were snap‐frozen in OCT solution and sectioned (10 µm‐thick slices) and mounted on microscope slides before being fixed in ice‐cold acetone and imaged using a Typhoon™ FLA7000 scanner (ex. 635 nm, band filter 670 nm; GE Healthcare Life Sciences, Chicago, IL). Following fluorescence imaging, the slides underwent IHC by staining with H&E (Leica Biosystems, Buffalo Grove, IL), anti‐affibody IgG mAb (1:50, AffibodyAB, Stockholm, Sweden) and anti‐EGFR mAb (1:200, Dako, Santa Clara, CA).

### Photoimmunotherapy *in vitro* studies

The cytotoxic effect of Z_EGFR:03115_–IR700DX‐based PIT *in vitro* was investigated using cells grown as 2D and 3D cell cultures. U87‐MGvIII or MCF7 cells (4 × 10^3^) were seeded in black 96‐well plates with clear bottoms for 24 h. For 3D U87‐MGvIII or WSz4 cultures, cells (4 × 10^3^) were seeded in 96‐well ultra‐low attachment plates (Corning® Costar®, Corning, NY) for 72 or 120 h (for WSz4). On the day of the experiment, fresh medium containing Z_EGFR:03115_–IR700DX (50 nM to 5 µM) or IR700DX (500 nM to 5 µM) was added for 6 h at 37°C. Cells or spheroids were then rinsed twice with phenol‐red‐free medium. Wells were irradiated in groups of nine using a red LED L690‐66‐60 (Marubeni, Tokyo, Japan). Further experimental details about light dosimetry are given in the Supporting Information. Cell response to PIT was evaluated by the CellTiter‐Glo® luminescent cell viability assay (Promega, Madison, WI) 24 or 96 h post‐irradiation in the 2D and 3D cell cultures. In addition, formation and growth of 3D spheroids was monitored daily by the Celigo® image cytometry system (Nexcelom Bioscience, Lawrence, MA).

### *In vivo* studies

Detailed treatment methods are described in the Supporting Information. All *in vivo* experiments were performed in compliance with licences issued under the UK Animals (Scientific Procedures) Act 1986 and following local ethical review. Studies were compliant with the United Kingdom National Cancer Research Institute Guidelines for Animal Welfare in Cancer Research.^25^ Female NCr athymic mice (6 weeks) were obtained from the in‐house breeding colony. Mice were inoculated subcutaneously (s.c.) in the top right shoulder with U87‐MGvIII cells (5 × 10^5^) resuspended in PBS and mixed with BD Matrigel™ Matrix (40%, v/v%, BD Matrigel™ Matrix, BD Bioscience, San Jose, CA). Details of the intracranial model are in the Supporting Information. Orthotopic brain tumors grew for 11 days, at which point MR and fluorescent imaging (for details see the Supporting Information) and brain extraction was performed.

### Statistical analyses

Unless otherwise stated, *in vitro* data were expressed as the mean ± SEM and *in vivo* as the mean ± SD. Statistical significance, sample size calculations and correlation analysis are described in detail in the Supporting Information.

## Results

### *In vitro* characterization of Z_EGFR:03115_–IR700DX

**Figure 1:**
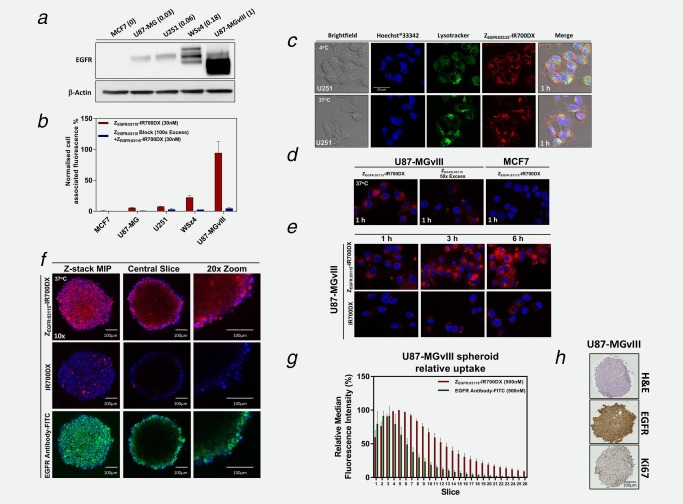
Expression of EGFR in GBM cell lines and specificity of the Z_EGFR:03115_–IR700DX binding to EGFR. (*a*) Varying EGFR expression in the selected cancer cell lines was confirmed by Western blot. The numbers in the brackets represent the relative EGFR expression as determined by densitometric analysis. (*b*) Z_EGFR:03115_–IR700DX (30 nM) binding as assessed by flow cytometry in the selected cancer cells with varying EGFR expression and after blocking with 100‐fold excess of unlabeled Z_EGFR:03115_. Data are presented as mean ± SEM (*n* = 3). (*c*–*f*) Confocal microscopy images demonstrating target‐specific binding (4°C) and internalization (37°C) of either the Z_EGFR:03115_–IR700DX (1 µM), anti‐EGFR‐FITC antibody (25 nM for visualization purposes) or IR700DX alone (1 µM): (*c*) U251 cell lines (1 h incubation time), (*d* + *e*) U87‐MGvIII or MCF7 cell lines (1–6 h incubation time), (*f*) U87‐MGvIII spheroids (6 h incubation time). Hoechst®33342 (blue) and LysoTracker™Green DND‐26 (green) were used for counterstaining. (*g*) Quantification of fluorescence intensity (median fluorescence intensity) of ∼8 µm slices through U87‐MGvIII spheroids following a 6 h incubation with Z_EGFR:03115_–IR700DX (500 nM) or an anti‐EGFR‐FITC antibody (500 nM). Data are presented as mean ± SEM (*n* = 3). (*h*) H&E, EGFR and Ki67 immunostaining of U87‐MGvIII spheroid (400–500 µm) sections 72 h after seeding.

EGFR expression in the selected GBM cancer cell lines and the breast cancer cell line, MCF7, was confirmed by Western blot (WB; Fig. 1 *a*). The corresponding densitometric analysis of protein bands indicated that EGFR expression level ranged from overexpression (U87‐MG_vIII_, 1), through intermediate (WSz4, 0.18) to very low (U251, 0.06; U87‐MG, 0.03) and negligible expression (MCF7, 0; Fig. 1 *a*). The EGFR‐specific (Z_EGFR:03115_) and non‐specific (Z_Taq_) affibody molecules were successfully conjugated via the maleimide group to IR700DX and the fluorescent‐SDS‐PAGE as well as silver staining confirmed labeling of the conjugates (Supporting Information Figs. 1*a* and 1*b*).

The specificity of Z_EGFR:03115_–IR700DX binding *in vitro* correlated with the expression level of EGFR as seen by flow cytometry and, importantly, pre‐blocking the cells with 100‐fold excess of unlabeled affibody molecules effectively reduced the median fluorescence in all tested cell lines (Fig. 1 *b*). Furthermore, confocal microscopy images showed intense binding of the conjugate to the cell membrane of U251 cells at 4°C, which further confirmed binding specificity of the Z_EGFR03115_–IR700DX to EGFR (Fig. 1 *c*, first row). At 37°C, however, the majority of the Z_EGFR03115_–IR700DX was uniformly distributed in the cytoplasm after 1 h (Fig. 1 *c*, second row, Fig. 1 *e*). Blocking cells with 100‐fold excess of unlabeled affibody resulted in an almost complete absence of fluorescence from the corresponding conjugate, confirming that the process is receptor mediated (Fig. 1 *d*). Additionally, as expected, MCF7 cells expressing very low levels of EGFR showed negligible fluorescent signal from the conjugate (Fig. 1 *d*). Cytoplasmic uptake was found to increase with incubation time, which indicated time‐dependent internalization of the conjugate (Fig. 1 *e*). In addition, co‐staining the cells with the conjugate and a lysosomal marker revealed significant colocalization (Fig. 1 *c*). Afterwards, in order to assess the penetration depth of the Z_EGFR:03115_–IR700DX, which is critical for successful PIT, 3D U87‐MGvIII or WSz4 spheroids that more closely resemble *in vivo* tissue in terms of cellular communication, were cultured with media containing either the conjugate, IR700DX or mAb_EGFR_‐FITC (Fig. 1 *f*, Supporting Information Fig. 2*b*, Supporting Information Movie 2–4). Importantly, Z_EGFR:03115_–IR700DX penetrated to a depth of around 200 μm after incubation for 6 h (Figs. 1 *f*, first row and 1*g*). Whereas, as expected, the antibody‐based conjugate was mostly restricted to the exterior layers (Figs. 1 *f*, third row and 1*g*), and hydrophilic IR700DX dye alone formed only a few fluorescent clusters on the very outer layer of the spheroid (Fig. 1 *e*, second row). Of note, by 72 h, H&E staining of the tumor spheres (400–500 µm) did not indicate any necrosis and positive nuclear staining for Ki‐67 confirmed that the relatively large inner cores of the spheroids still contained actively proliferating cells (Fig. 1 *h*, Supporting Information Fig. 2*a*).

### Z_EGFR:03115_–IR700DX‐mediated cell death *in vitro*

**Figure 2:**
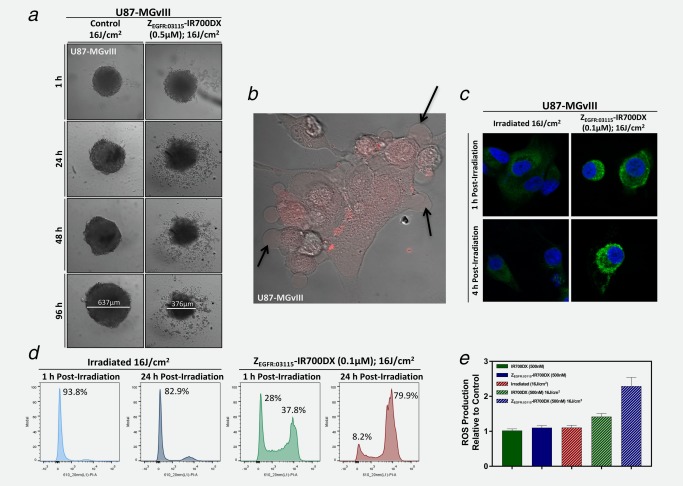
*In vitro* morphological changes following affibody‐based PIT. (*a*) Incubation of U87‐MGvIII spheroids with the Z_EGFR:03115_–IR700DX for 6 h and irradiation with a red LED (16 J/cm^2^) induced phototoxic cell death and disintegration of the architectural structure of the spheroid population. (*b*) U87‐MGvIII cells grown as a monolayer culture showed rapid cell swelling and bleb formation (see arrows) as visualized by a phase‐contrast image 1 h post Z_EGFR:03115_–IR700DX (red) irradiation with the 639 nm laser on a confocal microscope. (*c*) Following methanol fixation of U87‐MGvIII cells, either treated by PIT or just irradiated, and staining with an anti‐calreticulin‐AlexaFluor488 antibody overnight (4°C), images were acquired by confocal microscopy. (*d*) Cell membrane disruption was monitored by propidium iodide (1 µg/mL) staining. U87‐MGvIII cells irradiated only or treated with Z_EGFR:03115_–IR700DX‐based PIT were analyzed by flow cytometry 1 and 24 h post‐treatment. (*e*) Reactive oxygen species production was assessed using the DCFDA cellular ROS detection assay kit using U87‐MGvIII cells treated with affibody‐based PIT (15 min after light exposure). The results were normalized to the control cells. Data are presented as mean ± SEM (*n* = 3).

In order to capture cell morphologic changes induced by Z_EGFR:03115_–IR700DX‐based PIT, U87‐MGvIII and WSz4 spheroids were imaged using a Celigo® cytometer 1, 24, 48 and 96 h (Fig. 2 *a*) post‐continuous irradiation with the red LED L690‐66‐60 for 1,280 sec (16 J/cm^2^). Phase‐contrast images of cells grown in monolayers (2D) were taken using the Zeiss LSM700 confocal microscope to monitor the cells up to 2 h post‐irradiation with the 639 nm laser (5 mW; Fig. 2 *b*) and showed very rapid cell swelling and bleb formation shortly after irradiation (Fig. 2 *b*, Supporting Information Movie 1), indicating rapid cell death, most likely by necrosis. Similar effects were also observed for 3D spheroids, but more evidently at later time points (Fig. 2 *a*, Supporting Information Fig. 2*d*). Necrotic cell death was further confirmed by propidium iodide (PI) staining, demonstrating that cell membrane integrity had been disrupted as early as 1 h post‐irradiation (Fig. 2 *d*). Furthermore, light activation of Z_EGFR:03115_–IR700DX induced ROS generation (Fig. 2 *e*) that triggered cell death‐associated signals involved in immunogenic cell death leading to the apparent translocation of calreticulin to the cellular membrane (Fig. 2 *c*).

**Figure 3:**
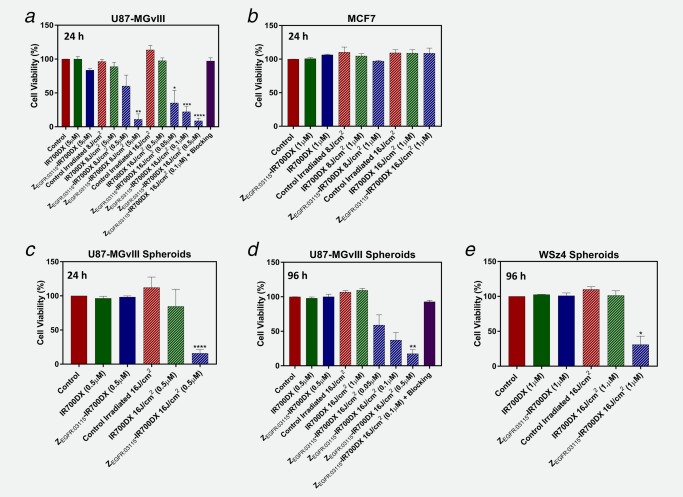
Z_EGFR:03115_–IR700DX‐mediated PIT causes cellular death selectively in EGFR+ve cells. Decrease in cell viability as assessed by the CellTiter‐Glo® luminescent cell viability assay 24 or 96 h post‐PIT in 2D cells and 3D spheroids, following 6 h incubation with the Z_EGFR:03115_–IR700DX and irradiation with a light dose of 8 or 16 J/cm^2^, was confirmed to be dose dependent and receptor mediated. (*a*) U87‐MGvIII cells 24 h post‐PIT. (*b*) MCF7 cells 24 h post‐PIT. (*c*, *d*) U87‐MGvIII spheroids 24 and 96 h post‐PIT. (*e*) WSz4 spheroids 96 h post‐PIT. Data are presented as mean ± SEM (*n* = 3). Statistical significance in comparison to the control group was determined using an unpaired two‐tailed Student's *t‐*test with Welch's correction. *p ≤ 0.05, **p ≤ 0.01, ***p ≤ 0.001 and ****p ≤ 0.0001. [Color figure can be viewed at http://wileyonlinelibrary.com]

To determine the phototoxicity of Z_EGFR:03115_–IR700DX, cells were incubated with the conjugate for 6 h and exposed to two different doses of NIR light. Cell viability of the 2D cultures was measured 24 h post‐treatment and after 24 and 96 h for 3D spheroids. The percentage of cell death in targeted cells was significantly influenced by the dose of excitation light (Fig. 3 *a*) and by the number of receptors (U87‐MGvIII+ve *vs*. MCF7‐ve; Figs. 3 *a* and 3 *b*). U87‐MGvIII cells treated with 500 nM of conjugate and irradiated with 16 J/cm^2^ demonstrated ∼90% decrease in cell viability 24 h post‐irradiation (CellTiter‐Glo® luminescent cell viability assay). In contrast, the treatment had no effect on the negative control (MCF7‐ve; Fig. 3 *b*). As expected, there was no significant cytotoxicity associated with exposure to Z_EGFR:03115_–IR700DX without NIR irradiation, IR700DX or light irradiation alone. Importantly, as shown in Figure 3 *a*, when cells were pre‐incubated with 100‐fold excess of non‐labeled affibody molecules, no cell death was observed, confirming that the phototoxicity is target specific. In light of these findings, cell viability following exposure to PIT was investigated in 3D cultures using U87‐MGvIII and WSz4 spheroids. The CellTiter‐Glo® luminescent assay again showed a significant and light dose‐dependent decrease in cell viability of U87‐MGvIII spheroids, with ∼85% of the cells dying 24 h post‐PIT treatment (500 nM of conjugate, 16 J/cm^2^ light exposure from the LED L690‐66‐60). WSz4 spheroid cultures were less sensitive than U87‐MGvIII spheroids (Fig. 3 *e*), presumably due to the lower EGFR density and likely a more resistant phenotype. In line with the results obtained for 2D cell cultures, there was no response when conjugate or light were used alone in either of the cell lines (Figs. 3 *c*–3 *e*).

### Imaging *in vivo* tumor targeting with Z_EGFR:03115_–IR700DX

**Figure 4:**
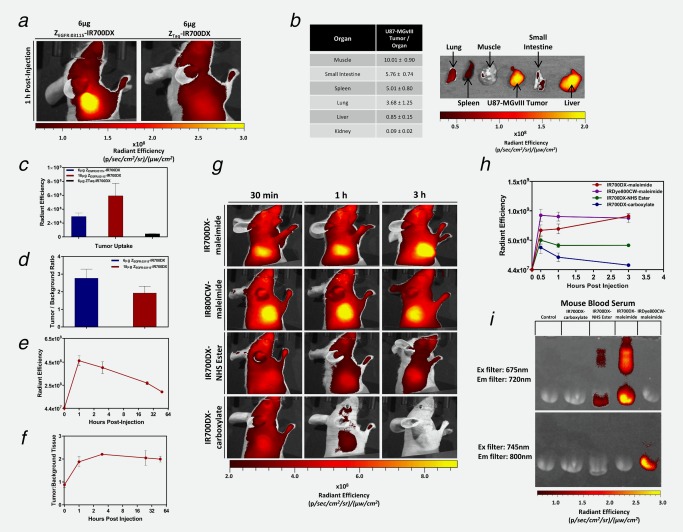
Testing Z_EGFR:03115_–IR700DX specificity *in vivo* and studying the effect of functional groups on dye pharmacokinetics. (*a*) The U87‐MGvIII tumor could easily be differentiated as early as 1 h post Z_EGFR:03115_–IR700DX (6 µg/mouse) being intravenously injected, whereas minimal tumor uptake was observed when administering the same amount of the non‐specific Z_Taq_‐IR700DX. (*b*) Fluorescence imaging of Z_EGFR:03115_–IR700DX uptake in excised tissues (1 h post‐injection) and respective tumor‐to‐organ ratios. (*c*) Mean radiant efficiency in U87‐MGvIII tumors 1 h after administering either 6 µg Z_EGFR:03115_–IR700DX, 18 µg Z_EGFR:03115_–IR700DX or 6 µg of the non‐specific Z_Taq_‐IR700DX. (*d*) Tumor‐to‐background ratio comparison when altering the injected dose of Z_EGFR:03115_–IR700DX. (*e*, *f*) Fluorescence intensity and tumor‐to‐background ratio in the U87‐MGvIII tumors over time after 18 µg Z_EGFR:03115_–IR700DX. (*g*, *h*) Fluorescence imaging of mice bearing subcutaneous U87‐MGvIII tumors. Images were acquired 30 min, 1 h or 3 h post IR700DX–maleimide, IR800CW–maleimide, IR700DX–NHS ester and IR700DX–carboxylate injection and the mean radiant efficiency was determined for each of the dyes. (*i*) An SDS‐PAGE gel of mouse blood serum imaged using the IVIS/Spectrum imaging system to visualize the fluorescent dyes’ association with blood proteins. All data are presented as mean ± SD (*n* ≥ 3).

The specific uptake of Z_EGFR:03115_–IR700DX *in vivo* was evaluated using mice bearing EGFR+ve subcutaneous U87‐MGvIII xenografts (Supporting Information Figs. 3*a* and 3*b*). When tumors reached 70–100 mm^3^, mice (*n* = 3) were injected with the conjugate (6 μg/mouse) or with the non‐specific affibody‐based conjugate Z_Taq_‐IR700DX (6 μg/mouse; Fig. 4 *a*, Supporting Information Fig. 3*a*). Due to high EGFR expression in normal tissues, for example, the liver and submaxillary salivary gland, 10 μg of non‐labeled Z_EGFR:03115_ was co‐injected with the conjugate in order to reduce off‐target EGFR binding. Subsequently, images were acquired 1 h post‐injection, the mice were sacrificed immediately afterward and their major organs extracted for further image analysis (Fig. 4 *b*). The tumor targeting by the Z_EGFR:03115_–IR700DX estimated from image ROIs showed a prominent fluorescence signal ((3.0 ± 0.43)×10^8^ p/sec/cm^2^/sr/μW/cm^2^) as early as 1 h post‐conjugate administration (Figs. 4 *b* and 4 *c*). The tumor signal intensity after Z_EGFR:03115_–IR700DX injection (6 μg) was calculated to be >6‐fold higher than the signal measured for Z_Taq_‐IR700DX, which confirmed the EGFR specificity of the affibody‐based conjugate *in vivo* (Figs. 4 *a* and 4 *c*). *Ex vivo* fluorescence images demonstrated that amongst the non‐targeted organs, the kidneys and liver exhibited the highest accumulation of the conjugate, producing tumor‐to‐organ ratios of 0.85 ± 0.15 and 0.09 ± 0.02, respectively (Fig. 4 *b*, Supporting Information Fig. 3*a*). This high renal accumulation is due to the glomerular filtration of the affibody molecules followed by protein reabsorption, degradation and retention in proximal tubular cells. The significant liver uptake is associated with high endogenous EGFR expression. Importantly, in contrast to radioisotope‐based imaging approaches, this does not predict toxicity because the conjugate is only cytotoxic within the very limited field in which NIR irradiation is delivered.

To determine the effect of increasing the conjugate dose, 18 µg of Z_EGFR:03115_–IR700DX was also administered which resulted in more than a twofold increase in tumor uptake (Fig. 4 *c*), although there was a decrease in tumor‐to‐background ratio (Fig. 4 *d*). Of note, tumor‐to‐background ratios are less important when the conjugate has no inherent toxicity of its own (in contrast to radioisotope‐based agents). Therefore, the conjugate dose of 18 µg was subsequently selected for PIT studies. To select the optimal therapeutic window for optical irradiation in the PIT study, we next evaluated the uptake of the conjugate at different time points. The fluorescence signal in the tumor was greatest 1 h post‐injection and then gradually decreased over time (Fig. 4 *e*), whereas the tumor‐to‐background ratios remained almost unchanged from 1 to 48 h (Fig. 4 *f*). Surprisingly, when we injected the equivalent to 18 µg Z_EGFR:03115_–IR700DX, of the highly hydrophilic IR700DX–maleimide, there was an intense tumor fluorescence that gradually increased over time and slow clearance was observed only after 24 h (Supporting Information Figs. 4*b* and 4*e*). Furthermore, *ex vivo* images of the ∼1.5 mm tumor sections confirmed deep penetration of the affibody molecules into the tumor tissue and high accumulation of the fluorophore (Supporting Information Fig. 4*d*). We hypothesized that this uptake was due to the reactive nature of the maleimide, so we compared the pharmacokinetics (PK) of IR700DX with three different functional groups: IR700DX–maleimide, IR700DX–NHS ester, IR700DX–carboxylate. Interestingly, tumor uptake was markedly lower when administering the IR700DX–NHS ester and IR700DX–carboxylate and was almost completely washed out after 3 h (Figs. 4 *g* and 4 *h*, Supporting Information Fig. 4*e*), indicating that the functional group was affecting the PK of IR700DX and the dye itself has no preferential uptake in the tumor. To further evaluate the influence of the maleimide linker, we also injected an equivalent dose of IR800CW–maleimide and found that the agent's behavior was very similar to the IR700DX–maleimide (Fig. 4 *g*, second row). We, therefore, postulated that the increased uptake of IR700DX–maleimide is due to an association with blood proteins, primarily blood serum albumin, via thiols which lead to longer systemic circulation and preferential accumulation in the tumor. To test our hypothesis, we analyzed mouse serum following administration of the respective dyes and discovered that the IR700DX–maleimide dyes appeared to have the highest affinity for the blood proteins based on the clear and intense fluorescent protein bands on the SDS‐PAGE gel (Fig. 4 *i*, Supporting Information Fig. 4*a*).

### Uptake of Z_EGFR:03115_–IR700DX in an orthotopic glioma model

**Figure 5:**
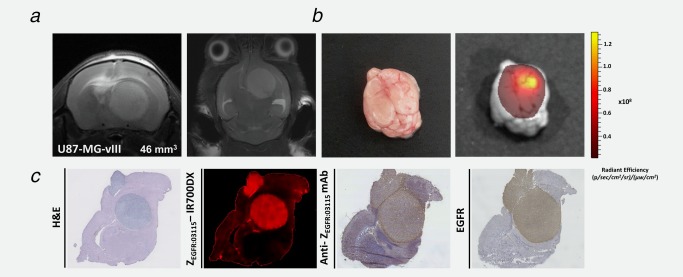
Z_EGFR:03115_–IR700DX accumulates in U87‐MGvIII orthotopic glioma tumors. (*a*) T_2_‐weighted MRI images of an intracranial brain tumor model 11 days post‐cell implantation. (*b*) Photographic image of the brain and the corresponding Z_EGFR:03115_–IR700DX fluorescent image demonstrates predominant accumulation of the conjugate within the brain tumor mass. (*c*) Transaxial brain histological sections (10μm) containing tumor tissue were obtained for *ex vivo* analysis immediately after 1 h *in vivo* image acquisition. Z_EGFR:03115_–IR700DX clearly delineated tumor mass from the surrounding normal tissues which correlated well with H&E and EGFR staining of the consecutive sections.

T_2_‐weighted images confirmed an intracranial tumor located in the right side of the brain of each mouse (Fig. 5 *a*). Tumor volumes determined from the MR images on the day of imaging, varied from 40 to 50 mm^3^ (*n* = 4). The fluorescence images acquired 1 h after i.v. injection of Z_EGFR:03115_–IR700DX (18 μg) revealed a fluorescent signal from the top of the skull (Supporting Information Fig. 5*a*). To confirm that the signal originated from the brain tumor, surgical excisions were performed to remove the brain which subsequently was imaged *ex vivo* (Fig. 5 *b*). An intense light signal was emitted from the site of tumor implantation (1.1 × 10^8^ p/sec/cm^2^/sr/μW/cm^2^) (Fig. 5 *b*). Calculated mean tumor‐to‐brain ratio was 16.6 ± 7.4 (*n* = 4; Supporting Information Fig. 5*b*). Although an equivalent dose of IR700DX–maleimide alone had higher tumor uptake (2.9 × 10^8^ p/sec/cm^2^/sr/μW/cm^2^), the surrounding brain had large amounts of non‐specific uptake and, therefore, only produced a mean tumor‐to‐brain ratio of 2.95 ± 1.07 (Supporting Information Fig. 5*b*). To further demonstrate that the signal following Z_EGFR:03115_–IR700DX administration correlated with EGFR expression, frozen brain sections were prepared, imaged by immunofluorescence and subjected to IHC. Distinct receptor expression correlated well with the histological staining of the tumor, the fluorescence image and IHC staining of Z_EGFR:03115_–IR700DX distribution. Importantly, minimal conjugate fluorescence was present in the surrounding mouse brain.

### Z_EGFR:03115_–IR700DX‐mediated PIT

**Figure 6:**
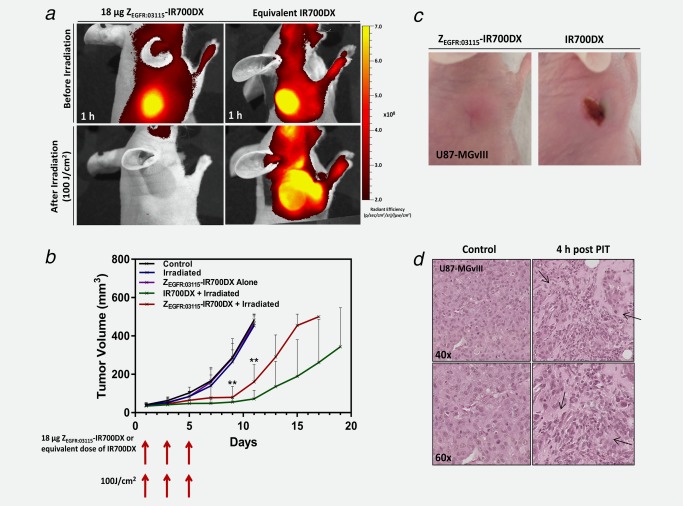
*In vivo* Z_EGFR:03115_–IR700DX‐mediated PIT studies. (*a*) Fluorescence imaging of mice bearing subcutaneous U87‐MGvIII tumors 1 h after injecting 18 μg of Z_EGFR:03115_–IR700DX or IR700DX–maleimide (top row). Subsequently, mice were irradiated with an optical dose of 100 J/cm^2^ by a red LED and, immediately after, imaged again (bottom row). (*b*) Tumor growth inhibition of the Z_EGFR:03115_–IR700DX‐targeted PIT in U87‐MGvIII tumors after administering three doses of 18 µg of the conjugate and irradiating with 100 J/cm^2^ at days 1, 3 and 5 in comparison to control groups. Data are presented as mean ± SD (*n* = 6 for each group, **p ≤ 0.01 as assessed by the Kruskal–Wallis test). (*c*) Visual observation of normal tissue damage in the PDT treated mice, while no skin damage was present in the Z_EGFR:03115_–IR700DX PIT mice. These were the appearances seen in all mice. (*d*) H&E staining of treated and untreated U87‐MGvIIII tumors (arrows indicate regions of tissue necrosis).

For the PIT studies, mice bearing subcutaneous U87‐MGvIII tumors were randomized into four groups (as in the Material and Methods section) and injected with Z_EGFR:03115_–IR700DX or IR700DX–maleimide. Following 1 h pre‐treatment image acquisition, the mice received a light dose of 100 J/cm^2^ using the LED L690‐66‐60. Figure 6 *a* clearly demonstrates that this dose was sufficient to photobleach the fluorescence signal, decreasing tumor fluorescence almost to background in the case of the conjugate. Interestingly, the IR700DX–maleimide fluorescence intensity not only did not photobleach, but rather increased in the tissues surrounding the tumor. This may be due to vessel dilation in response to irradiation with the NIR light and subsequent reaccumulation of the IR700DX in the tumor.

In total, each mouse received three PIT cycles, once every other day. There was a significant inhibition of tumor growth in mice treated with the Z_EGFR:03115_–IR700DX‐based PIT, as well as IR700DX‐based PDT, in comparison to the control groups that received only light or each of the agents alone (*p* < 0.01; control groups *vs*. PIT) over a period of 18 days (Fig. 6 *b*). Of note, control groups had to be sacrificed earlier (∼Day 11) because the tumor sizes approached the limits stipulated in institutional guidelines and animal project licence. The advantage of PIT over PDT was highlighted as mice treated with the IR700DX‐based PDT started to developed skin necrosis on the treated lesion, as early as 1 day after the first light exposure, whereas in PIT‐treated mice the normal tissue was spared (Fig. 6 *c*). Histopathological staining of tumor sections with H&E confirmed that the U87‐MGvIII control tumors had intact tumor morphology, whereas tumors treated with a single dose of PIT (18 µg Z_EGFR:03115_–IR700DX and an optical dose of 100 J/cm^2^) showed numerous regions of tissue necrosis (cells appeared to be sparse with less intense nuclei staining and vacuolar degradation), 4 h post‐irradiation (Fig. 6 *d*).

## Discussion

Considering the risk of recurrence of GBM if tumor cells remain following resection, any improvement in the intraoperative approaches that help to differentiate between tissue types and eradicate remaining tumor cells could substantially improve outcomes for GBM patients. To address this challenging problem, we evaluated an IR700DX affibody‐based conjugate (Z_EGFR:03115_–IR700DX) for image‐guided PIT using an EGFR+ve xenograft model of GBM. The rationale behind developing this particular conjugate came from recent findings showing that various disease‐specific NIR fluorophore‐based mAb probes (e.g., IR800CW–cetuximab) have been developed for FGS of GBM.^26^ However, the relatively large molecular size of mAbs may limit their extravasation into the GBM tumor, especially in areas with only partial BBB disruption. Additionally, mAbs may not reach glioma cells that have migrated beyond the main tumor mass. Therefore, Sexton *et al*. and others, have recently investigated EGFR‐specific affibody molecules, that are ∼20 times smaller than mAbs, labeled with IR800CW and revealed that the probe can successfully detect glioma margins.^21^, ^27^, ^28^, ^29^ Interestingly, it was reported that there is a significantly higher accumulation of Affibody_EGFR_–IR800CW than cetuximab–IR680RD in the boundaries of glioma tumor, even though cetuximab has ∼30 times greater affinity for EGFR than affibody molecules.^27^ Building on these findings, we went one step further and conjugated Z_EGFR:03115_ affibody molecules with the highly hydrophilic IR700DX via a maleimide group. Of note, IR700DX‐mAb‐based PIT targeting EGFR has been previously demonstrated to be effective in cancers such as breast, lung and bladder by Kobayashi and co‐workers.^30^, ^31^, ^32^, ^33^, ^34^ We found that Z_EGFR:03115_–IR700DX has significant activity in inducing cell death selectively in EGFR+ve GBM cells both *in vitro* and *in vivo*. NIR light irradiation of U87‐MGvIII cells following incubation with the conjugate effectively induced cellular death in a receptor‐mediated manner. No toxicity was observed when the cells were treated with the Z_EGFR:03115_–IR700DX or IR700DX alone.

We then asked whether exposure of Z_EGFR:03115_–IR700DX to NIR light results in cell membrane disruption shortly after treatment, since it has been previously reported that PIT leads to rapid necrotic cell death.^15^, ^18^ Consistent with previous studies, we observed prominent cellular swelling, bleb formation and release of cellular content from U87‐MGvIII cells within 1 h post‐treatment initiation which suggested necrotic cell death. Complementary to this, at 1 h 37.8% of cells subjected to both light exposure and the conjugate showed positive PI staining, confirming that the integrity of the cellular membrane was compromised.

Similarly to our *in vitro* data with Z_EGFR:03115_–IR700DX, *in vivo* imaging studies demonstrated that the conjugate penetrates deeply in the EGFR highly expressing U87‐MGvIII subcutaneous and orthotopic tumors, allowing for clear tumor visualization as early as 1 h post‐injection. In contrast, intact antibodies require at least a day to achieve a high tumor‐to‐background ratio due to a high blood background, which could prove problematic for pre‐surgery administration. Moreover, there was only negligible tumor uptake when mice were injected with the non‐specific Z_Taq_‐IR700DX affibody molecules that confirmed *in vivo* specificity of the conjugate. Importantly, the *in vivo* PIT experiments showed significant differences in tumor growth between U87‐MGvIII tumor‐bearing mice treated with the conjugate and control groups: (*i*) mice that were optically irradiated only and (*ii*) mice that received Z_EGFR:03115_–IR700DX or saline only. While a promising start, these findings require further in‐depth validation studies using orthotopic PDX glioma models that more precisely recapitulate the histopathological properties and maintain genomic characteristics of parental GBMs *in situ*. It will be challenging, but orthotopic GBM brain tumors underneath the intact skin and skull have recently been unambiguously visualized using the enhanced NIR fluorescent protein IFP2.0.^35^ In addition, Jing *et al*. have reported that CD133‐IR700DX mAb‐based NIR‐PIT extended the overall survival of mice with patient‐derived orthotopic gliomas by a factor of two when light was applied through the skull following i.v. administration of the conjugate.^36^

We also questioned whether IR700DX alone induces any antitumor effects. Surprisingly, and in contrast to our *in vitro* experiments, we showed that IR700DX rapidly penetrated into the tumor mass and, when exposed to NIR light, inhibited tumor growth. Intrigued by these findings, we compared the PK of IR700DX with three different functionalities: (*i*) maleimide (required for affibody conjugation), (*ii*) *N*‐hydroxysuccinimide (NHS) ester (the most frequently used functional group for mAb conjugation), and (*iii*) carboxylate. All of them demonstrated distinctly different PK behaviors. The IR700DX–NHS ester and IR700DX–carboxylate had their highest uptake in the U87‐MGvIII tumor at 30 min post‐injection and were cleared from the tumor faster than the IR700DX–maleimide. This could be attributed to the highly hydrolysable nature of the NHS group in aqueous solution which, *in vivo*, may be catalysed by hydrolytic enzymes present in various tissues and plasma. Conversely, maleimide functionalities may adhere to blood proteins with active thiol‐groups remaining in the body much longer. These results were in agreement with previous evidence showing that the linker moiety can affect the PK of the probe.^37^ Even though the IR700DX–maleimide was highly effective and has the potential to be a useful non‐targeted PS *in vivo*, its high non‐specific binding to serum proteins, post‐treatment skin toxicity and lack of tumor specificity will limit the probe's utility especially for intraoperative PDT of brain tumors.

In conclusion, GBM is characterized by a heterogeneous expression of amplified and mutated EGFR which presents a substantial challenge for the effective use of EGFR‐directed therapies. Recently, several clinical trials using first‐ and second‐generation PSs have demonstrated promising results when used as PDT agents targeting GBM, but have not yet been approved for routine clinical practice. This may be attributed to the lack of specific cancer‐targeting properties of these PSs and poor optical activation sensitivity, which is particularly important at greater tissue depth. Encouragingly, our *in vitro* and proof‐of‐concept *in vivo* studies clearly highlight the potential of eradicating EGFR+ve glioma cells using Z_EGFR:03115_–IR700DX‐targeted PIT. The conjugate benefits from the small molecular weight of its targeting moiety, high specificity of binding to EGFR (wild‐type and EGFRvIII), and emission of NIR fluorescence that permits high imaging resolution with increased tissue penetration depth (>2 cm). These results are particularly exciting in light of recent findings suggesting that EGFR amplification and mutation are initial events in the pathogenesis of GBM and that anti‐EGFR treatments might be effective as an early therapeutic intervention.^38^ While EGFRvIII‐positive GBM cells may only represent a small percentage of total population of cancer cells, as lately demonstrated, they can be responsible for the survival of non‐EGFRvIII‐expressing tumor cells and the evasion of molecularly targeted systemic therapy regimens.^39^ Therefore, killing EGFRvIII‐positive “linchpin” cells using intraoperative EGFRvIII‐based PIT may alter the complex behavior of the microscopic residual tumor cells, resulting in better outcomes for GBM patients.

## Supporting information

Supporting Information Movie 1

Supporting Information Movie 2

Supporting Information Movie 3

Supporting Information Movie 4

Supporting Information
