# Supplementary material for: Near‐infrared photoimmunotherapy targeting EGFR—Shedding new light on glioblastoma treatment
Source: Int J Cancer. 2018 Jan 19;142(11):2363–74. doi: 10.1002/ijc.31246 (PMC6016485; doi:10.1002/ijc.31246)
Supplement: Supplementary file 5 — Supporting Information [file IJC-142-2363-s005.doc]

Supplementary Information:

Title: Near-Infrared Photoimmunotherapy Targeting EGFR - Shedding New Light on Glioblastoma Treatment

**Authors:**

Thomas A. Burley^1^, Justyna Mączyńska^1^, Anant Shah^1^, Wojciech Szopa^2^, Kevin J. Harrington^1^, Jessica K.R. Boult^1^, Anna Mrozek-Wilczkiewicz^3^, Maria Vinci^4^, Jeffrey C. Bamber^1^, Wojciech Kaspera^2*^ & Gabriela Kramer-Marek^1*^

**Affiliations:**

^1^Division of Radiotherapy and Imaging, The Institute of Cancer Research, London, UK

^2^Department of Neurosurgery, Medical University of Silesia, Regional Hospital, Sosnowiec, Poland

^3^A. Chelkowski Institute of Physics, University of Silesia, Katowice, Poland,

^4^Department of Onco-Hematology, Bambino Gesù Children’s Hospital, Rome, Italy

**Contents:**

**Materials and Methods:**

**Conjugation of the affibody molecules with IR700DX**

The Z_EGFR:03115_ or Z_Taq_ (250 µg, 37.2 nmol) were first reduced with a 20-fold molar excess of Tris(2-carboxyethyl)phosphine (TCEP) in phosphate buffer pH 7.2 for 5 min at 85°C, and then 25 min at room temperature (RT). Next, the reduced Z_EGFR:03115_ was incubated with a nine-fold molar excess of IRDye700DX-maleimide (648 µg, 327.4 nmol) for 2 h at 40°C. After conjugation, the sample was purified by twice passing the sample through a Zeba™ Spin Desalting Column (7K MWCO, 2 mL) (Thermo Fisher Scientific). The concentration of the conjugate was determined by measuring the absorbance at 689 nm using a NanoDrop™ 2000 spectrophotometer (Thermo Fisher Scientific). The yield of the Z_EGFR:03115_–IR700DX conjugation was found to be 35.9 ± 2.6 % due to loss of affibody during purification through the Zeba column. The calculated Degree of Labeling (DOL) for Z_EGFR:03115_–IR700DX and Z_Taq_-IR700DX were 0.84 and 0.92 moles of dye per mole of affibody, respectively. HPLC was not performed due to the highly pH sensitive nature of the dye. The purity of the conjugate was confirmed by first running the samples by SDS-PAGE, with non-labeled Z_EGFR:03115_ affibody molecules being used as a control, staining the proteins with a Silver Staining kit, according to the manufacturer’s protocol (Thermo Scientific), and imaging the gel using a ChemiDoc™XRS System (Bio-Rad). The fluorescence bands were directly visualized using a Typhoon™ FLA7000 scanner (ex. 635 nm, band filter 670 nm; GE Healthcare Life Sciences).

**Cell lines and cell culture**

Patient tumor sample was collected fresh at the time of surgery from the operation theatre at the Department of Neurosurgery, Regional Hospital in Sosnowiec, Poland, in accordance with the Institutional Ethical Committee approval. Tumor was classified as GBM based on the WHO criteria by examination of a registered pathologist (Supplementary Information Fig. 6). Part of the surgical sample was minced and live cryopreserved in DMEM:F12, 0.2% BSA, 10% DMSO. The WSz4 cell line was established upon mechanical dissociation from cryopreserved tissue following the procedures previously reported and cells were grown as an adherent culture (two-dimensional, 2D) on flasks coated with laminin (Sigma) under stem cell conditions (neurobasal medium was supplemented with N2 and B27 supplements (Thermo Fisher Scientific, Invitrogen); 20 ng/mL of each recombinant human growth factors bFGF and EGF; and additionally 2 μg/mL Heparin (StemCell Technologies), GlutaMAX™-I and antibiotic-antimycotic solution (Life Technologies). The genetic origin of these cells was tested and authenticated by short tandem repeat (STR) DNA profiling analysis (Eurofins Medigenomix, Germany). Results were compared with STR profiles published online at https://www.dsmz.de/services/services-human-and-animal-cell-lines/online-str-analysis.html, matching for the following markers: amelogenin, CSF1PO, D16S539, D5S818, D7S820, TH01, TPOX, D13S317 and vWA. EGFR expression of WSz4 patient-derived cell line was confirmed by IHC (Supplementary Information Fig. 2C).

**Western blotting**

Rabbit monoclonal antibodies against EGFR (1:1000) and β-Actin (1:1000) were purchased from Cell Signaling Technology. The blots were incubated overnight at 4^o^C with the indicated primary antibodies, followed by a 1 h incubation at RT with horseradish peroxidase-conjugated secondary antibody and subsequent detection using an enhanced chemiluminescence reagent (Pierce). Finally, the membranes were scanned using a ChemiDoc™ XRS+ System (Bio-Rad). Densitometric analysis was performed using ImageJ software with protein levels being normalized to the β-Actin loading controls. The central band was used for the quantification of WSz4 and the lower band for the U87-MGvIII.

**Flow cytometry**

To verify the target specificity of the Z_EGFR:03115_-IR700DX, *in vitro* cell binding studies were conducted by flow cytometry. Approximately 3x10^5^ cells from each cell line were incubated with 30 nM Z_EGFR:03115_-IR700DX for 1 h at 4°C with or without 100-fold excess of unlabeled Z_EGFR:03115_ affibody molecules. After incubation with the affibody, cells were washed twice with cold phosphate-buffered saline (PBS) and resuspended in 400 µL of PBS for data acquisition. Flow cytometry was performed using the BD™ LSRII flow cytometer (BD Biosciences). For each sample, 10,000 events were recorded, and the population corresponding to single cells was gated and analyzed as a histogram plot. Three independent experiments were performed and each point was normalized to the median fluorescence intensity acquired for U87-MGvIII cells.

To assess the level of cell membrane disruption, U87-MGvIII cells were first incubated with 100 nM Z_EGFR:03115_-IR700DX for 6 h and irradiated with 16 J/cm^2^ (see below for dosimetry) using a red LED L690-66-60 (Marubeni). Next, 1 or 24 h post irradiation, propidium iodide (1 µg/mL) was added for 15 mins at RT and cells were subsequently analyzed by the BD™ flow cytometry (LSRII BD Biosciences).

**Confocal microscopy**

To test the specificity of the conjugate binding Z_EGFR:03115_-IR700DX (1 µM) or IR700DX alone (1 µM) were added to the media and the cells were incubated for 1, 3 or 6 h at 37°C. Additionally, one group of U87-MGvIII cells was pre-incubated with 50-fold excess of the non-labelled Z_EGFR:03115_ molecules 10 min before adding the Z_EGFR:03115_-IR700DX. Also, U251 cells were incubated with the conjugate for 1 h at 4°C to minimize receptor internalization prior to microscopy. To analyze the penetration of the conjugate in comparison to an antibody-based conjugate, U87-MGvIII spheroids were incubated with either Z_EGFR:03115_-IR700DX (1 µM), anti-EGFR targeted Antibody-FITC (25 nM for visualization / 500 nM for quantification) or IR700DX-Maleimide alone (1 µM). To observe calreticulin translocation, U87-MGvIII cells were fixed with 100% methanol at -20°C, blocked with 5% bovine serum albumin and incubated overnight with anti-calreticulin antibody conjugated to Alexa Fluor®488 (Abcam). For counterstaining, cells were exposed to Hoechst®33342 (nuclear stain; 5 µg/mL) and/or Lysotracker™ Green DND-26 (lysosome stain; 5 nM) 1 h before imaging (both from Thermo Fisher Scientific). Images were captured using the Zeiss LSM700 confocal microscope (Carl Zeiss Inc) equipped with a 40x (numerical aperture, NA - 1.3) Plan-Apochromat oil immersion objective and a 10x (numerical aperture, NA - 0.45) Plan NeoFluar air objective. Hoechst®33342, Lysotracker™ Green DND-26, Alexa Fluor®488 and IR700DX- fluorescence signals were visualized by excitation with 405 nm, 488nm and 639 nm laser lines, respectively. Images were analyzed using the Zen 2009 software (Zeiss).

**Photoimmunotherapy *in vitro* studies**

PIT experiments were done using a red LED L690‑66‑60 (Marubeni) that provides continuous light (peak wavelength 690 nm, bandwidth 670‑710 nm) with a mean intensity of 12.5 mW/cm^2^ (LED current = 275 mA) for a duration of either 640 s or 1280 s, resulting in a mean optical fluence of either 8 or 16 J/cm^2^, respectively. Optical dosimetry was assisted by using plates with black-walled wells, and involved measuring the total power delivered per well using a power meter (PM160T, Thorlabs) placed under a well that had its plastic base removed, and a mask that allowed only light that had passed through that well to reach the detector. The measured power was then divided by the cross-sectional area of the well to obtain the intensity. This was carried out for each of the four wells in a group, and the mean intensity calculated. The coefficient of variation (ratio of standard deviation to mean) of light dose over the four wells was approximately 17% of the mean. To assess ROS production following PIT U87-MGvIII cells were seeded into a 96 well plate (15x10^3^ per well) overnight. The cells were incubated with Z_EGFR:03115-_IR700DX (500 nM), IR700DX (500 nM) or media alone for 6 h at 37°C before following the protocol as described in the DCFDA - Cellular Reactive Oxygen Species Detection Assay Kit (Abcam). After delivering to the cells the light dose of 16 J/cm^2^, the fluorescence was measured using a FLUOstar Omega microplate reader (ex. filter: 485 nm, em. filter: 520 nm).

***In vivo* studies**

Tumors were measured every other day and their volume was calculated according to the formula: 1/2(Length × Width^2^). Tumors had reached an approximate volume of 70-100 mm^3^ before initiating imaging studies or 40-70 mm^3^ for the Z_EGFR:03115_-IR700DX-based PIT treatment studies. The Z_EGFR:03115-_IR700DX (6µg or 18µg), IR700DX-carboxylate, IR700DX-NHS ester, IR700DX-maleimide and the IR800DyeCW-maleimide were injected intravenously (i.v.) and images were acquired at the indicated time points using the IVIS/Spectrum/CT imaging system (PerkinElmer) (ex. filter: 675 nm, em. filter: 720 nm). Afterwards, regions of interest (ROIs) were drawn around the tumors and background tissue, radiant efficiency images (images in units of photons/sec/cm^2^/sr that have been divided by a stored image of the excitation light intensity in units of µW/cm^2^) were taken and the average radiant efficiency in each ROI was calculated. To study the pharmacokinetics (PK) of the Z_EGFR:03115-_IR700DX after 1 h, tissues were excised, mean fluorescence was recorded and the tumor-organ ratios were determined, allowing selection of the optimal conditions for the following PIT studies. For the treatment studies, 6 mice were randomly assigned to each group: (i) no treatment; (ii) light exposure only (100 J/cm^2^); (iii) 18 µg Z_EGFR:03115_-IR700DX injected by i.v. without light exposure; (iv) 18 µg Z_EGFR:03115_-IR700DX injected by i.v. with light exposure (100 J/cm^2^); (v) equivalent dose of IR700DX-maleimide injected by i.v. with light exposure (100 J/cm^2^). The surrounding normal tissues were protected with aluminum foil during irradiation and the light was delivered to the tumor for a duration of 516 seconds by placing the LED L690‑66‑60 2 to 3 millimeters above the tumor (intensity measured at the skin surface = 0.194 ± 0.012 W/cm^2^, LED current of 300 mA). Animals were monitored daily and the tumor volume recorded every other day. When the tumors exceeded 500 mm^3^ the mice were sacrificed.

**Intracranial model**

For the intracranial model, U87-MGvIII cells (1x10^5^) were resuspended in PBS and kept on ice until injection. To perform intracranial stereotactic implantation of cells, mice were anaesthetized using 1.5%-2% isoflurane mixed with oxygen. The scalp was then swabbed with alcohol, and a small incision in the skin was made to expose the bregma. A small (1.0 mm) burr hole was drilled at 1 mm anterior and 2 mm lateral to the bregma. A 10-μl syringe was used to slowly inoculate 5 μl of cell suspension at a depth of 2.5 mm from the dura. Afterwards, the needle was slowly withdrawn, the incision was glued and appropriate local and systemic analgesia was given. Brain tumors grew for 11 days, at which point imaging and brain extraction were performed.

**MRI imaging**

MRI was performed at day 11 post implantation on a horizontal bore Bruker Biospec 70/20 (Ettlingen, Germany) equipped with physiological monitoring equipment (SA Instruments, Stony Brook, NY, USA) using a 2 cm x 2 cm mouse brain array coil. Following optimization of the magnetic field homogeneity using a localized map shim over the whole brain, a rapid acquisition with relaxation enhancement (RARE) T_2_-weighted sequence (repetition time (T_R_) = 4500 ms, effective echo time (T_Eeff_) = 36 ms, 4 averages, RARE factor = 8, in-plane resolution 98 µm x 98 µm, 1 mm thick contiguous axial and coronal slices) was used for localization of the tumor and measurement of tumor volume.

**Statistical analyses**

Statistical significance was determined using unpaired two-tailed Student’s *t* tests with Welch’s correction or assessed by the Kruskal-Wallis test. Six mice per time point were allocated for *in vivo* assessment of Z_EGFR:03115_-IR700DX. The sample size was calculated with 90% power and 5% significance level. The mice were randomized before treatment initiation. No data were excluded from the analysis, including all outliers. Three experimental replicates were performed for each *in vitro* experiment. Statistical significance was determined using Prism software (GraphPad Software v7.0). Statistically significant differences between groups were assumed if *P* ≤ 0.05.

**Supplementary Figures:**

**
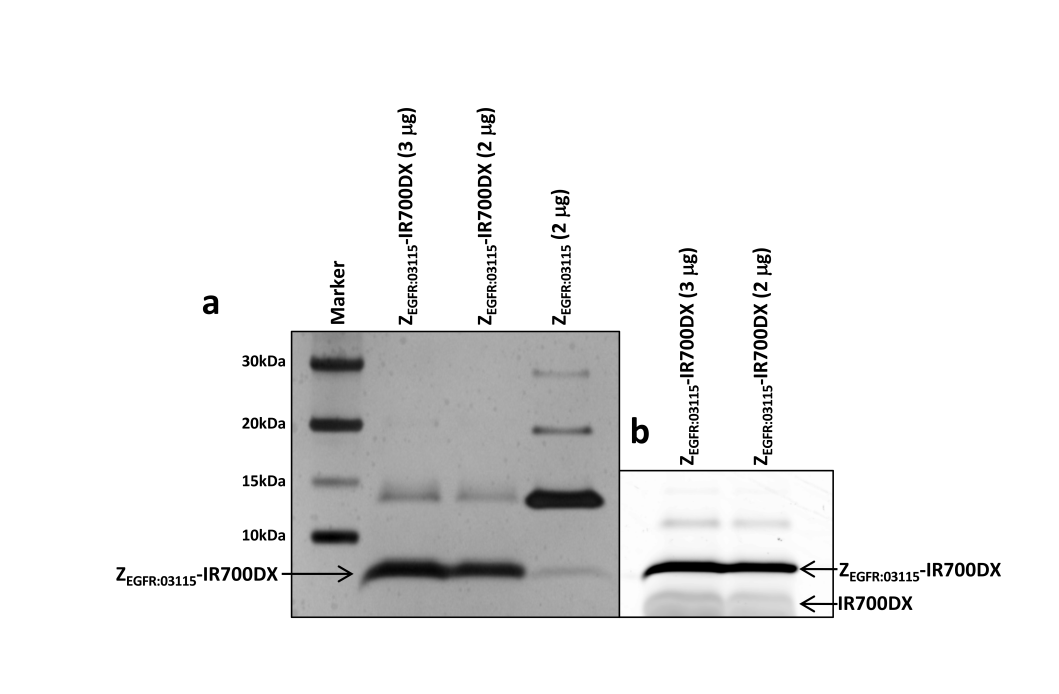
**


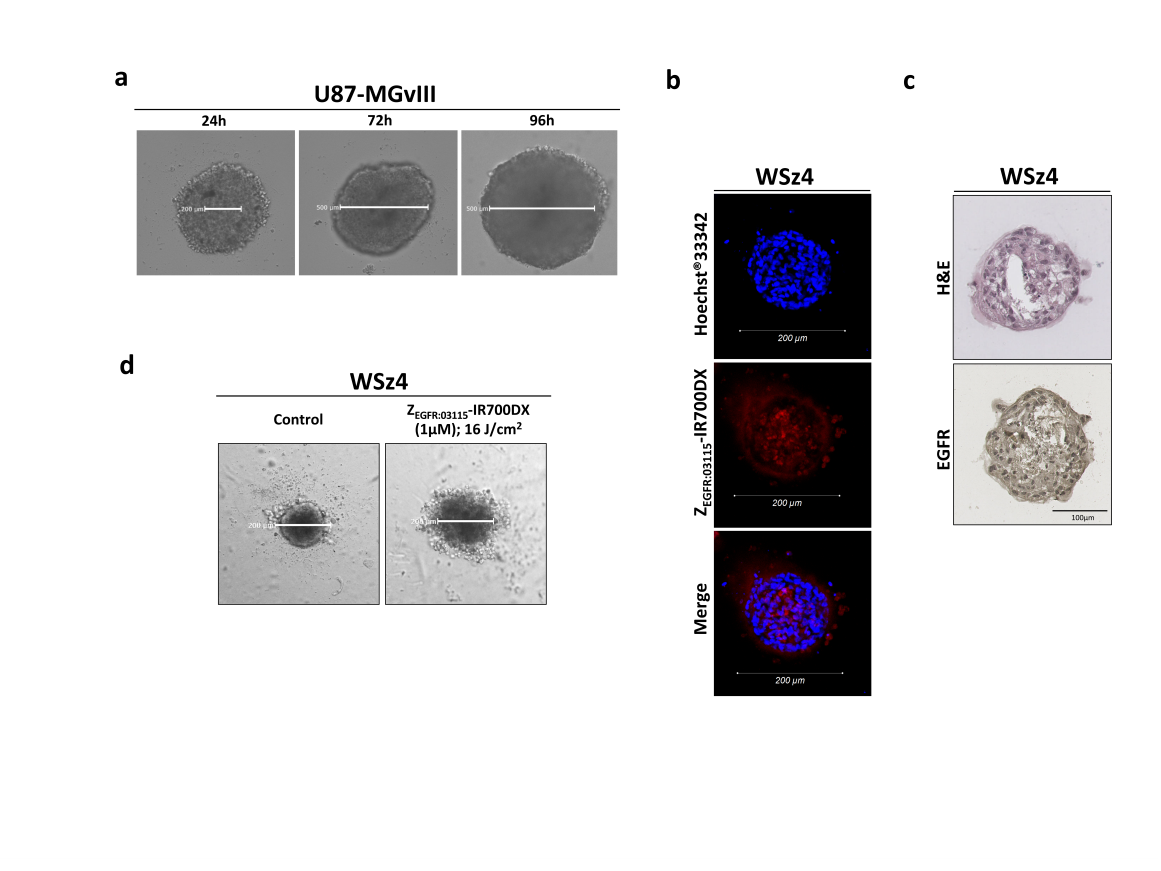
**Supplementary Information Fig. 1. (a)** Silver staining of the gel to ensure reduction was achieved and the quantification of the conjugate was accurate. **(b)** Fluorescence image of the conjugated Z_EGFR:03115_-IR700DX separated by Tricine SDS-PAGE which confirmed that the conjugation was successful, with minimal presence of IR700DX.

**Supplementary Information Fig. 2. (a)** U87-MGvIII spheroid growth over time. Representative images were captured using the Celigo® image cytometry system at indicated time points. **(b)** Z-stack MIP of a WSz4 spheroids following a 6 h incubation with either Z_EGFR:03115_-IR700DX (Red) (1 µM) and Hoechst®33342 (Blue). **(c)** Immunohistochemistry H&E staining and staining for EGFR in WSz4 spheroid sections. **(d)** Morphological changes in a WSz4 spheroid 96 h after PIT in comparison to a control spheroid.

**
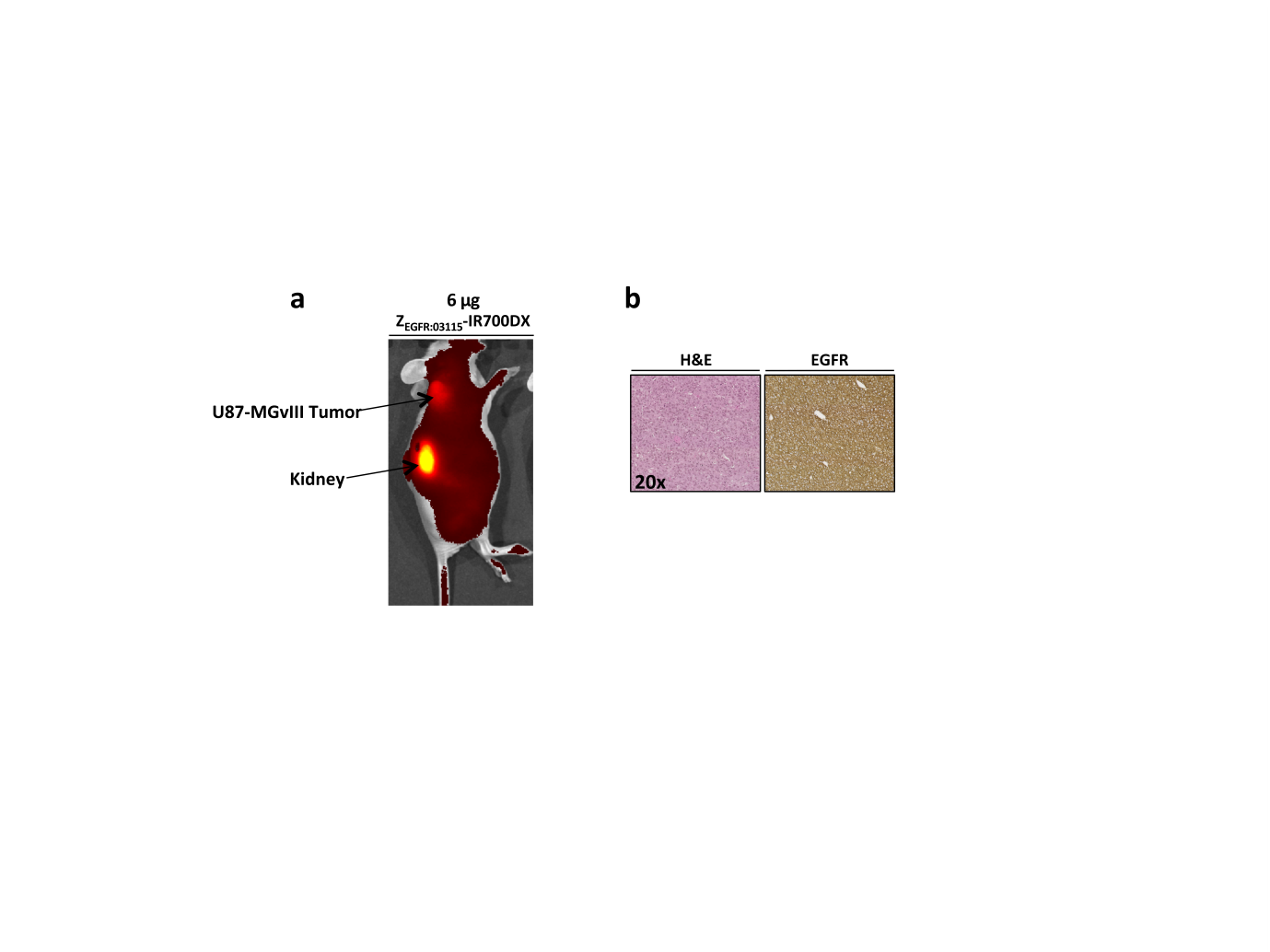
**

**Supplementary Information Fig. 3. (a)** Lateral whole body image 1 h after 6 µg Z_EGFR:03115_-IR700DX injection. **(b)** H&E and EGFR immunostaining of U87-MGvIIII tumors.

**
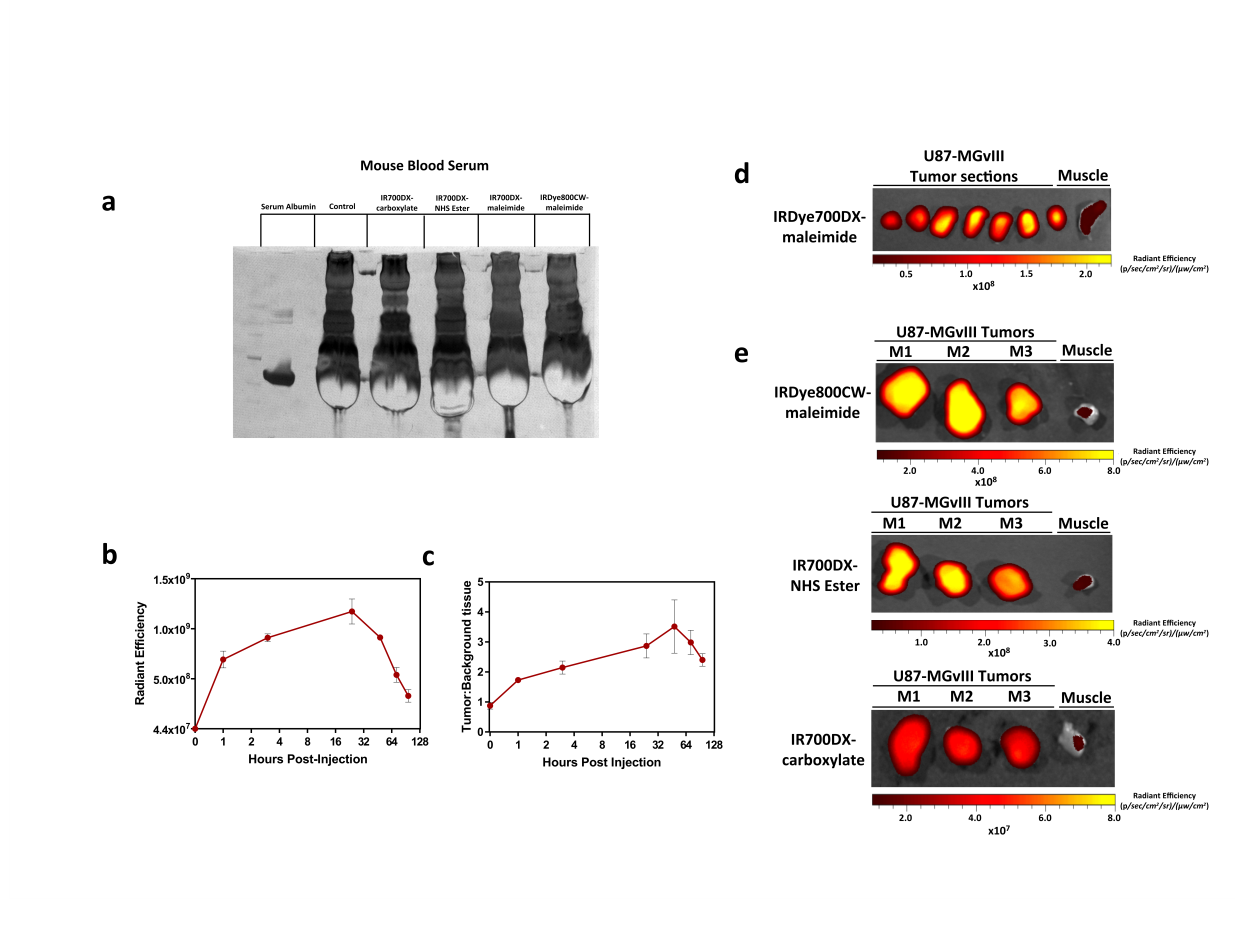
**

**Supplementary Information Fig. 4. (a)** Silver stain of blood serum run alongside pure serum albumin on SDS-PAGE. (**b and c)** Fluorescence intensity and tumor-to-background ratio in U87-MGvIII tumors over time after an equivalent to 18 µg Z_EGFR:03115_-IR700DX of IR700DX-maleimide was injected. **(d)** U87-MGvIII tumor excised and sliced 1 h post-injection of 2.07 nmol IR700DX-maleimide (equivalent to 18 µg Z_EGFR:03115_-IR700DX) alongside a slice of muscle. **(e)** Varying dye uptake in excised U87-MGvIII tumors 3 h post-injection of 2.07 nmol of each dye (equivalent to 18 µg Z_EGFR:03115_-IR700DX) in comparison to muscle.

**
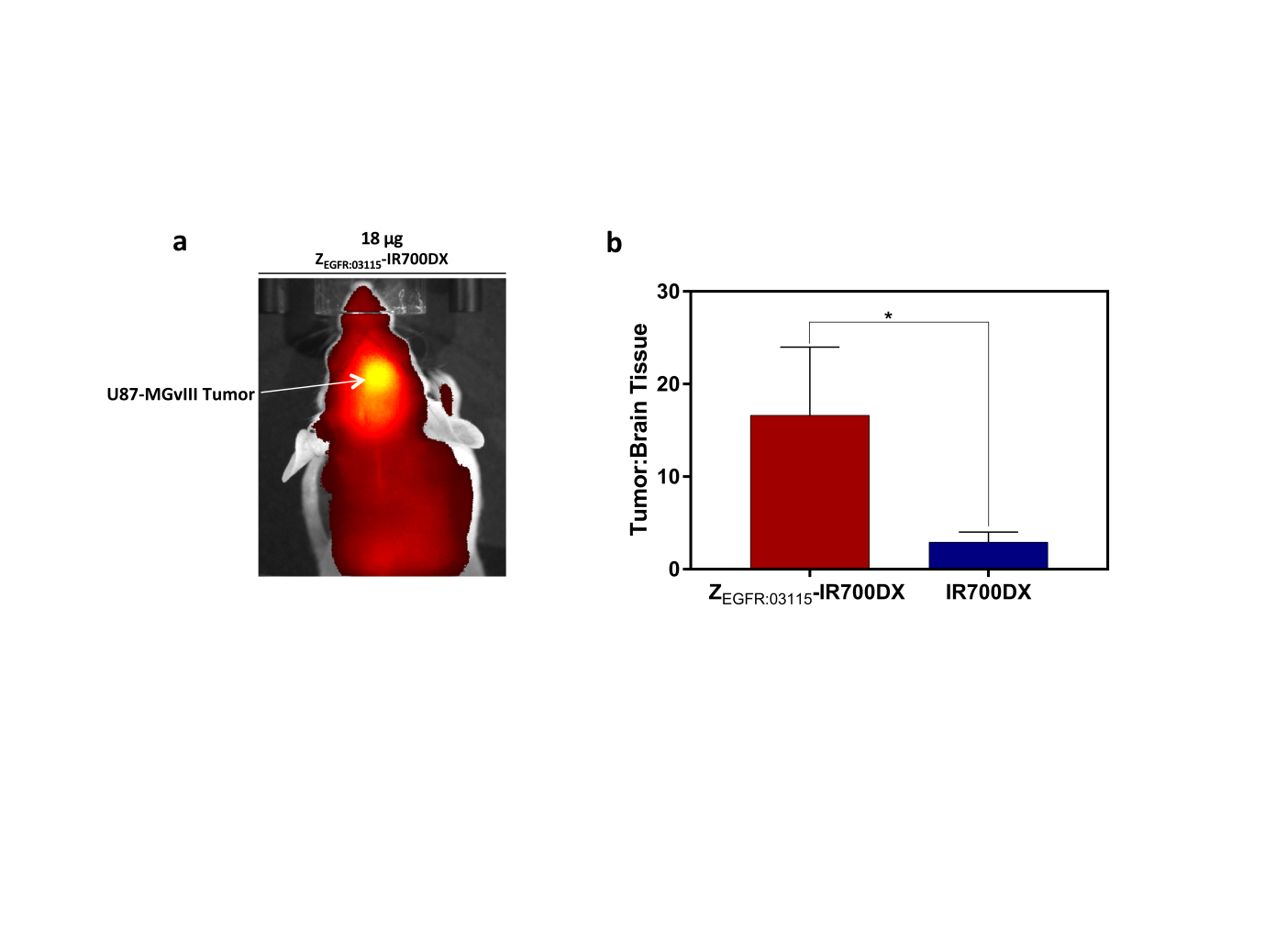
**

**Supplementary Information Fig. 5. (a)** *In vivo* fluorescence imaging of Z_EGFR:03115_-IR700DX uptake by intracranial brain tumor 1 h after conjugate injection. **(b**) The ratio of tumor uptake to normal brain tissue uptake was determined by quantification of the excised brain images following intravenous injection of 18 μg of Z_EGFR:03115_-IR700DX or equivalent IR700DX-maleimide. Data are presented as mean ± SD (n ≥ 3)

**
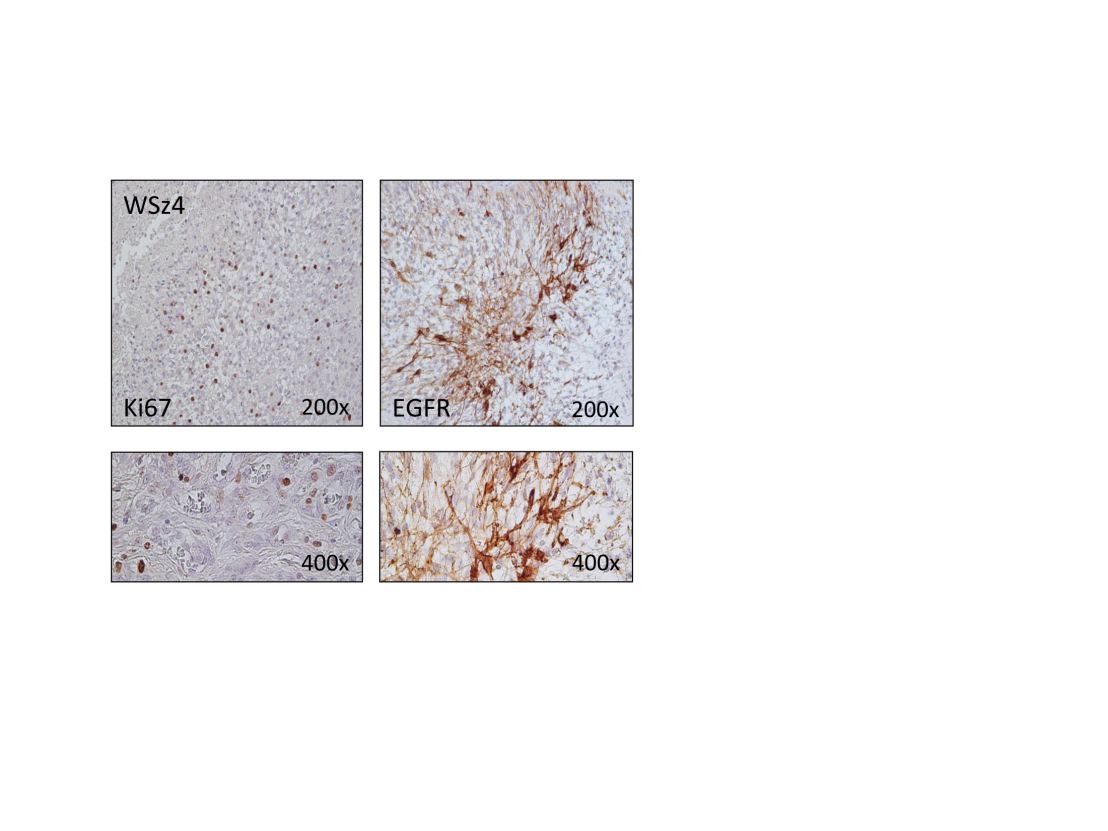
**

**Supplementary Information Fig 6.** 30 yr old man was diagnosed with a secondary GBM (WHO IV). Tissue obtained during surgery was stained with H&E and anti-EGFR. The primary cell line from the frozen sample has been recently established in our lab (WSz4).

**Supplementary Movies:**

**Movie S1**

U87-MGvIII cells grown as a monolayer culture have shown rapid cell swelling and bleb formation as visualized by a phase contrast time lapse over a period of 2 h following Z_EGFR:03115_-IR700DX (red) irradiation with the 639 nm laser on a confocal microscope.

**Movie S2, S3, S4**

Videos of sequential Z-stack images of U87-MGvIII spheroids following a 6 h incubation with either Z_EGFR:03115_-IR700DX (Red, 1µM) (Movie 2) or EGFR Antibody-FITC (Green, 25 nM) (Movie 3) and
a WSz4 spheroid stained with Z_EGFR:03115_-IR700DX (Red, 1µM) (Movie 4). All cells were counterstained with Hoechst®33342 (Blue).

**References:**

1. Pollard SM, Yoshikawa K, Clarke ID, Danovi D, Stricker S, Russell R, et al. Glioma Stem Cell Lines Expanded in Adherent Culture Have Tumor-Specific Phenotypes and Are Suitable for Chemical and Genetic Screens. Cell Stem Cell. 2009;4:568-80.
